# Development of neural perceptual vowel spaces during the first year of life

**DOI:** 10.1038/s41598-019-55085-y

**Published:** 2019-12-20

**Authors:** Kathleen M. McCarthy, Katrin Skoruppa, Paul Iverson

**Affiliations:** 1 Department of Linguistics, Queen Mary University of London, Mile End Road, London, E1 4NS United Kingdom; 2 Institut des sciences logopédiques, Université de Neuchâtel, Rue Pierre-à-Mazel 7, 2000 Neuchâtel, Switzerland; 3 Speech, Hearing and Phonetic Sciences, University College London, Chandler House, 2 Wakefield Street, London, WC1N 1PF United Kingdom

**Keywords:** Language, Perception

## Abstract

This study measured infants’ neural responses for spectral changes between all pairs of a set of English vowels. In contrast to previous methods that only allow for the assessment of a few phonetic contrasts, we present a new method that allows us to assess changes in spectral sensitivity across the entire vowel space and create two-dimensional perceptual maps of the infants’ vowel development. Infants aged four to eleven months were played long series of concatenated vowels, and the neural response to each vowel change was assessed using the Acoustic Change Complex (ACC) from EEG recordings. The results demonstrated that the youngest infants’ responses more closely reflected the acoustic differences between the vowel pairs and reflected higher weight to first-formant variation. Older infants had less acoustically driven responses that seemed a result of selective increases in sensitivity for phonetically similar vowels. The results suggest that phonetic development may involve a perceptual warping for confusable vowels rather than uniform learning, as well as an overall increasing sensitivity to higher-frequency acoustic information.

## Introduction

The standard account of speech development has been that it begins *in utero*, with newborns demonstrating learning for the prosodic elements of speech that could be heard before birth^1,2^. In contrast, the perception of higher-frequency aspects of speech (vowels and consonants) has been thought to be language-universal at birth, and through exposure to speech acoustics of the ambient language, babies begin to become specialised for their native vowels and consonants in the second half of the first year of life, which seems to facilitate later word learning^3–5^.

This summary, however, hides some complexity in the actual developmental trajectory, which has become particularly apparent in recent research. For example, vowel and word learning have now been shown to begin earlier; there is some evidence of language-specific vowel perception shortly after birth based on the low-frequency acoustic information transmitted to the womb^6^, and evidence that word-form learning may begin as early as 4.5 months of age (^7^; c.f^8,9^). There is also evidence that infants show a particular sensitivity to lower-frequency acoustic cues. For example, early vowel and word learning has been claimed to be more dependent on the lowest vowel resonant frequencies (F1) than higher vowel resonances (^10,11^; see also^12^), and that vowel learning may occur earlier than for higher-frequency consonant cues (^3^; cf^13^). We still do not fully understand the interplay of acoustic environment, hearing maturation, and the development of speech perception in the first year of life.

Charting developmental patterns in speech perception has been difficult, in part, because traditional methods only allow for the assessment of few phonetic contrasts in a single experiment (e.g., [i]-[ɪ] as in *sheep-ship*). This problem can partly be addressed via meta-analyses (e.g.^14,15^), but such analyses do not allow for a direct comparison of abilities across the vowel space. Our goal here was to develop a method to test as many vowel pairs as possible in a single experiment, allowing us to better view the pattern of learning across the entire vowel space, and how this pattern changes throughout the first year of life. To do so, we used the Acoustic Change Complex (ACC) of EEG recordings. The ACC is elicited to an acoustic change in an ongoing sound (e.g., amplitude or spectral change among vowels or consonants), and is thought to be generated by the primary auditory cortex (see^16–18^). In adults, it unfolds over time with multiple peaks (P1-N1-P2), and the later peaks can be affected by attention and learning, rather than being purely driven by auditory sensitivity^19–21^. Critical for the present study, the ACC can be measured using long concatenated vowel sequences with multiple vowel category changes per second, which has been used for time-efficient hearing assessments in clinical situations for adults and children^22,23^. Here we used this time-efficiency to present many vowel pairs in a single experiment (random sequences of seven British English vowels, creating 42 different transitions between vowels, i.e., 21 vowel pairs in two orders. See Results Fig. 2) and tested 83 monolingual English infants aged four to eleven months old. We analysed to what extent these ACC responses were driven by low- and high-frequency spectral differences among the vowels, and used multidimensional scaling^24,25^ to map how sensitivity to these vowel contrasts changed with age.

**Figure 1:**
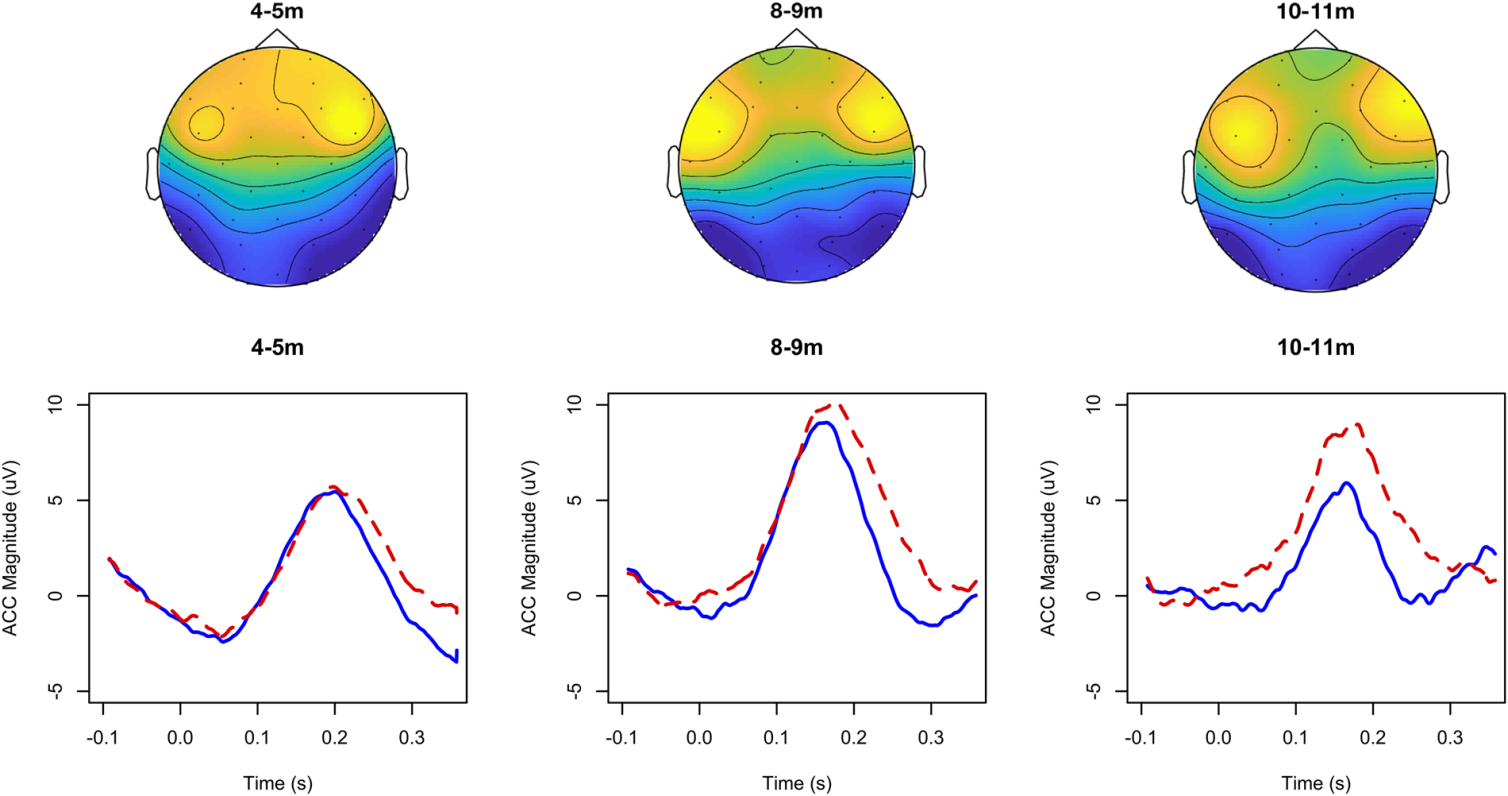
Sensor-space heatmaps for the peak ACC response, and the average ACC response for the vowel pair /ɪ/-/ɒ/ at the highest-response electrode. Blue lines indicate spectral changes in the order of /ɪ/-/ɒ/, and red dashed lines indicate /ɒ/-/ɪ/. The ACC response for these stimuli had a frontal-central, and slightly bilateral, scalp distribution. All ages had a peak around 200 ms, with the latency decreasing somewhat with age. There were also some small order asymmetries, in this case with a larger response in the /ɒ/-/ɪ/ direction.

## Results

Infants each completed an average of 2879 trials (i.e., concatenated vowel pairs; average of 137 trials per vowel pair) during a testing session that lasted an average of eighteen minutes. Figure 1 shows scalp distributions for the ACC and an example mean ACC response for the vowel pair /ɪ/-/ɒ/. The ACC within this paradigm was a bilateral frontal-central response, with a prominent peak approximately 200 ms after the sound change. Figure 1 displays boxplots of the ACC magnitude for each pair. The magnitude of the ACC varied with the acoustic difference between vowels (e.g., small response for /i/-/u/), and with changes in the ACC response with age.

**Figure 2:**
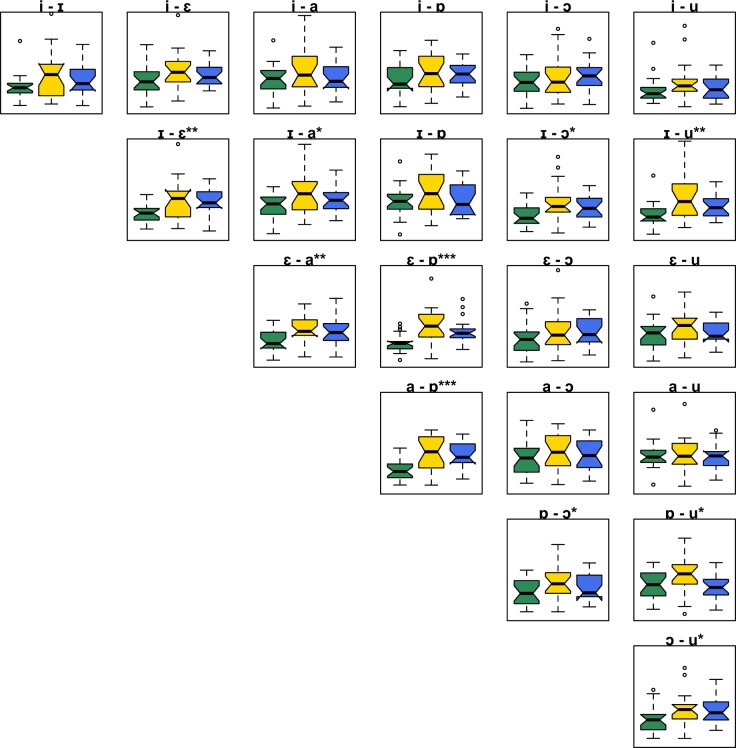
Box plots of maximum ACC values for each vowel pair across the three age groups: 4–5 month-olds (green), 8–9 month-olds (yellow) and 10–11 moth olds (blue). The y-axis represents microvolts, ranging from 0–25 μV. The notches represent 95% confidence intervals. The asterisks refer to the *p* values (*p < 0.05, **p < 0.01, ***p < 0.001) for the one-way ANOVAS comparing the max ACC across the three age groups for the named vowel pair. See Supplementary Material for the full ANOVA tables.

This variation in the ACC was evaluated by a linear mixed-effects model with the magnitude of the ACC as the dependent variable, age group and vowel pair as categorical fixed factors, and by-subject random intercepts; the factors were evaluated within a Type II analysis-of-variance table. This analysis tested whether there were significant effects overall before additional tests on individual pairs, as this age x pair interaction is complex to interpret on its own. There were significant main effects of vowel pair (*χ*^2^(20) = 208.53, *p* < 0.001) and age (*χ*^2^(2 = 7.18, *p* = 0.027) and a significant interaction (*χ*^2^(40) = 75.93, *p* < 0.001). Given this significant interaction, a set of orthogonal one-way ANOVAs were conducted for each vowel pair, comparing the magnitude of the ACC across the three age groups in order to evaluate the change with age for each vowel pair; Fig. 2 displays the *p* values for each pair (see Supplementary Material for full ANOVA tables). Significant increases in sensitivity (i.e., a larger ACC response) across the three age groups tended to be found for nearby vowel pairs along the vowel quadrilateral (e.g., /ɪ/-/ɛ/), and were less often present for more distant vowel pairs. This suggests that infants may selectively increase their sensitivity through development to the individual vowel pairs that are phonetically most difficult to distinguish, rather than globally increasing their sensitivity for vowels or increasing their sensitivity to all pairs involving particular vowels (e.g., point vowels such as /i/).

### Relationship of ACC development to speech acoustics

A linear mixed-effects model similar to above, with the cochlear-scaled spectral difference between vowel pairs replacing the “pair” fixed factor, demonstrated that the ACC responses became progressively less driven by acoustics through development. The cochlear-scaled spectral difference quantified the overall acoustic dissimilarity between pairs of vowels, with frequency and amplitude on a scale that approximates human hearing^26^. There was a significant interaction between spectral difference and age (*χ*^2^(2)= 16.03, *p* < 0.001), with the relationship between acoustic difference and ACC magnitude being strongest at the younger ages. There were also significant main effects of age (*χ*^2^(2) = 7.18, *p* = 0.027), and spectral difference (*χ*^2^(1) = 54.39, *p* < 0.001).

**Figure 3:**
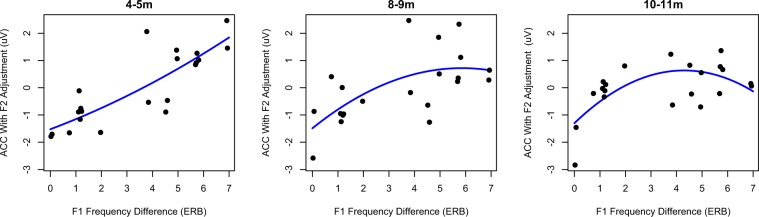
Relationship between F1 frequency difference and ACC magnitude for each pair, with the effect of F2 on ACC magnitude removed through regression. At 4–5 months, there is a linear relationship between ACC magnitude and F1 frequency difference. However, by 10–11 months infants have reduced ACC magnitude only for the smallest two F1 differences; at higher F1 differences there was little relationship between the magnitude of the difference and the ACC. That is, the ACC becomes less related to raw F1 differences through development.

A further analysis, with the first and second formant frequency differences entered as fixed factors in place of cochlear-scaled spectral difference, demonstrated that the interaction of spectral change with age was related mostly to F1 frequency. That is, there was a significant interaction between F1 frequency difference and age (*χ*^2^(2)= 13.64, *p* = 0.001). Figure 3 displays the average ACC magnitude for each pair as a function of F1 difference, with the effect of F2 variation removed through regression. The youngest babies had increasing ACC magnitude with increasing F1 difference, but the oldest group had a curvilinear relationship with ACC magnitude flattening out as F1 differences became large. There were likewise main effects of age (*χ*^2^(2) = 7.18, *p* = 0.028), F1 difference (*χ*^2^(1) = 115.81, *p* < 0.001), and F2 difference (*χ*^2^(1) = 13.17, *p* < 0.001), but no other significant interactions (*p* > 0.05).

### Age related changes in vowel perception maps

**Figure 4:**
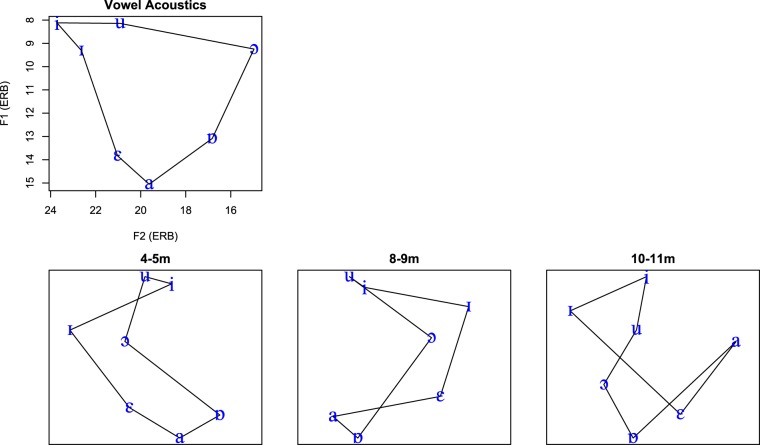
Formant frequencies of the vowels and the MDS solutions for vowels based on ACC magnitude for each age group. Vowels were placed close in the MDS space if there was a small ACC for that pair, and far apart for a larger ACC. Lines join the vowels that are adjacent along the vowel quadrilateral. The results demonstrate that the ACC responses at the youngest ages were mostly driven by F1; the vertical dimension corresponds to F1 but the front-back distinction related to F2 is not as clear on the horizontal dimension. At older ages, acoustically neighbouring pairs of vowels begin to have disproportionately large ACC responses (e.g., /a/-/ɒ/ at 10–11 months), causing the MDS solutions to distort.

The perceptual vowel spaces were visualised by using classical multidimensional scaling (MDS, 24, 25) to fit the magnitude of the infants’ ACC responses to two-dimensional Euclidean spaces (Fig. 4), with pairs placed farther apart when they produced a larger ACC. Distances in the MDS solutions produced good fits to the data, modelling 95, 80, and 81% of the mean ACC magnitudes respectively for the three age groups. Consistent with the mixed-model analyses, the map for 4–5 month-olds somewhat resembled a traditional vowel quadrilateral, with the vertical high-low dimension relating to the lowest formant (F1) and less separation between vowels on the front-back horizontal dimension relating to the second formant (F2). At the older ages, however, the MDS solutions began to less resemble a vowel quadrilateral, with some neighbouring vowel pairs (e.g., /a/-/ɒ/) increasing in ACC magnitudes out of proportion with their relatively small acoustic differences.

### Order asymmetries

Given that each vowel pair was presented to the infants in both presentation orders (e.g., /i/-/ɪ/ and /ɪ/-/i/), we were able to investigate asymmetries in the infants’ response. A linear mixed-effects model was conducted with asymmetry magnitude as the dependent variable, age group and vowel pair as fixed factors, and by-subject random intercepts. There was a main effect of pair (*χ*^2^(20) = 139.30, *p* < 0.001), but no significant main effect of age or an interaction (*p* > 0.05). Order asymmetries were mostly observed for vowel pairs that included vowels with a low first formant (i.e., /ɔ/ and /i/). In particular, the infants displayed a larger ACC when /ɔ/ was the second sound in the vowel pair. This pattern was present in all three age groups.

## Discussion

The present study measured neural sensitivity to acoustic changes among a large set of vowel pairs spanning the English vowel space, with the results demonstrating that the neural responses at younger ages are more acoustically driven and correspond more strongly to the first formant frequency, then become progressively less related to raw acoustic differences through development. This change in responses appears to occur around the age (e.g., 6 months) at which behavioural research^3^ have begun to find language-specific patterns of vowel perception. Although we cannot make language-specific claims without cross-linguistic data, the increases in sensitivity that we see from eight months old may be the result of the infants’ emerging native language phonology and growing lexicon (see e.g.,^3,4^). One novel aspect of our results is that we are not finding that ACC responses are increasing in development uniformly for all vowel pairs or increasing overall for particular vowel categories (e.g., point vowels). Rather, we are finding selective increases in sensitivity for nearby vowel pairs. It may be that the initial stages of vowel category learning involve dissimilating nearby acoustically confusable vowels, a process similar to perceptual warping^27,28^. That is, we likely didn’t find uniform increases in sensitivity across the vowel space with age because acoustically distant vowels are easily distinguishable without learning, whereas nearby vowels can benefit from a perceptual enhancement of acoustic differences to facilitate making categorical distinctions at later stages of the learning process (e.g.^27,28^).

Our finding of higher weighting of F1 acoustic variation is consistent with some previous findings on individual vowel pairs, but demonstrates that this weighting extends across the vowel space. For example, Lacerda^11^ found that 2–3 and 6–12 month-old infants were unable to discriminate a synthetic vowel contrast when the primary cue was the second formant (i.e., /ɑ/-/a/), but successfully discriminated contrasts that differed in their F1 dimension (i.e., /ɑ/-/ʌ/). This F1 sensitivity has also been found during the beginnings of word learning in older infants. For example, Curtin and colleagues^10^ found that 15 month-old infants were able to detect a switch in word-object pairing when the vowel contrast was in the F1 dimension (i.e., /ɪ/-/i/) but not in the F2 dimension (i.e., /i/-/u/ and /ɪ/-/u/). The new aspect of the present study is that this pattern of F1 sensitivity holds across a wide range of vowels, particularly at younger ages.

It is tempting to link this pervasive higher weighting for F1 with the fact that these babies would have primarily been exposed to low-frequency acoustic differences in the womb (e.g., <500 Hz). The basic idea that phonetic learning starts in utero is by no means novel; previous research on newborns has shown that exposure in utero shapes sensitivity to the prosodic elements of speech (^1,2^, see also^29^) and more recently for native language vowel contrasts (e.g.,^6^). Likewise, theories of phonetic acquisition, such as PRIMIR and NLM-e (^4,30^; see also^31^) incorporate differential weighting of acoustic cues and the role of prenatal experience into their models. However, interpreting the effects of F1 are complex; F1 has effects across the entire speech spectrum rather than only at low frequencies (e.g., low F1 frequencies produce vowels with less higher-frequency energy) and these correlated changes across the spectrum make it difficult to determine what individual frequencies were driving the ACC response. Moreover, this greater F1 weighting was clearly present in our youngest ages, but it was difficult to know whether this weighting decreased with age or whether the neural response became less related to acoustic differences overall. It is thus provocative that this high F1 sensitivity may be explained by in utero exposure, but we do not have clear proof.

We also found order asymmetries in our data. Previous infant work has also found asymmetries, both behaviourally and using MMN (e.g.^32,33^). Polka and colleagues (e.g.^32,33^) have concluded that extremes of the vowel space, where there are strong spectral peaks, act as perceptual attractors; a change from a more central to peripheral vowel is easier to discriminate than the opposite direction. Our results were broadly similar, in the sense that we found that vowels with strong low-frequency peaks (particularly /ɔ/) produced a larger ACC response when it followed a more central vowel. However, the asymmetries that we found in the ACC were not particularly large or systematic across the vowel space, and we didn’t find the age-related decline in asymmetries that has been found with the measures used in previous studies^32^. Interestingly, these results align with the conclusions drawn by Tsuji and Cristia^15^, whose meta-analysis also revealed peripherality effects, but failed to show age-related changes.

To summarize, the current study presents a detailed view of vowel perceptual development, revealing a more complex acquisition processes than previous methodologies have been able to explore. This efficient technique enables the tracking of multiple phonetic contrasts at the same time, from infancy to adulthood. Future research can utilise this method to investigate cross-linguistics differences across the lifespan, in both typical and atypical populations.

## Method

### Subjects

Eighty-three full-term monolingual English infants aged 4;0–4;30 (N = 27), 8;0–8;30 (N = 26) and 10;0–11;0 months (N = 30) were tested. To ensure enough data was collected per contrast for the analysis, infants with less than 1800 trials were excluded. As a result, twenty-three infants were excluded from the final analysis. A remaining twenty-two 4–5 month olds (*M* trials = 2882), twenty 8–9 month olds (*M* trials = 2836), and eighteen 10–11 month olds (*M* trials = 2492) were included in the analysis. The higher attrition rates in the eldest group was primarily due to the additional difficulties in testing mobile infants. All infants had no reported developmental delay and had passed the UK newborn hearing screen. The study was conducted with approval and under the accordance of the relevant guidelines established by the University College London Research Ethics Committee (2696/002). We obtained informed consent from the infants’ caregivers at the beginning of the lab visit.

### Stimuli

The stimuli were /i/, /ɪ/, /ɛ/, /a/, /ɒ/, /ɔ/, and /u/, as in the words *beat, bit, bet, bat, Bart, bought, and boot*, produced by a female native speaker of British English; all recordings had 44.1 kHz 16-bit/sample. The vowels were based on spoken sustained utterances that were intended to reduce spectral change (i.e., produced for more than a second) and then further processed to limit pitch and amplitude modulations. The sustained recordings were edited to select a 1.5 s portion of each vowel with little spectral change. An overlap-add method^26^ was used to flatten the pitch contour to an average value for the talker (185 Hz). The amplitude envelope of each recording was flattened by calculating the envelope of the original recording (full rectification and a 50-Hz low-pass filter), dividing the original recording by this envelope, and rescaling the RMS amplitude to be equal across vowels. See Supplementary Material Table 2 for the spectral difference measurements for each vowel pair. The sequences were created by splicing random sequences of vowels; each vowel was a 300–400 ms random segment from the longer recording, spliced with 50 ms of overlap (raised cosine window). See Supplementary Material for a sample of the stimuli.

### Apparatus

EEG recordings were made using a 16-channel (initial 11 subjects: five 4–5 month olds, six 10–11 month olds) and 32-channel (remaining 49 subjects) Biosemi Active Two system with three additional external electrodes (left and right mastoids, and horizontal EOG for eye movements). Recordings were made with a sampling frequency of 2048 Hz. Triggers were recorded via pulses on a disused audio channel that were converted to TTL triggers using a custom circuit.

### Procedure

During the EEG recordings, infants sat on their caregiver’s lap in a sound-attenuated booth. The stimuli were played to the infants as they watched a silent child-friendly video and were entertained by a research assistant with puppets and toys, to minimize movement artefacts. The caregivers could hear the stimuli, however given the passive nature of the task we expect no influence from the caregiver. The stimuli were presented at 68 dB SPL, measured across the stimulus sequences rather than for individual pairs. Each session lasted approximately 1.5 h, including preparation, breaks, and clean-up. The EEG recording lasted an average of 18 minutes.

### Processing and analysis

The recordings were referenced to the mastoid average. The lower back channels (O1, Oz, O2, P3, Pz, P4 for 16-channel system, plus PO3, PO4, P7 and P8 for the 32-chanel system) were not included in the analysis due to artefacts caused by head movements.

The continuous EEG recording was high-pass filtered at 0.1 Hz and low-pass filtered at 30 Hz using Butterworth filters as implemented in the ERPlab library^34^ of EEGlab^35^. Fieldtrip^36^ was used to interpolate any remaining noisy channels. The recordings were epoched with 100 ms pre-stimulus and 350 ms post-stimulus intervals, subtracting the baseline average over the pre-stimulus interval. Denoising Source Separation^37^ was used to extract a single ACC source for each subject, based on a procedure that selected the linear combination of the channels that maximized the repeatability of the average ACC response on individual trials. The response was then taken as the projection of this component back into sensor space at the electrode with the maximum amplitude; this scaled the DSS component back to EEG levels (uV). Following these steps, epochs were artefact-rejected if they had values outside of a ± 150 μV range, with the remaining epochs averaged. The ACC amplitudes were averaged in the 0–350 ms time window.

Mixed-model analyses used the lmer function in the R package lme4^38^, and the reported significance values were from a type II analysis-of-variance table calculated from the lmer models using the package CAR^39^. MDS solutions were calculated using the classical-MDS procedure cmdscale within R^40^, using two dimensions and including a constant. The plotted MDS solutions were translated, rotated, reflected, and scaled to promote the visual comparability of the solutions (i.e., plotting the vowel space with the orientation of a traditional vowel quadrilateral), as MDS solutions only display the relative similarity of items (i.e., the orientation, reflection, and scaling are arbitrary).

## Supplementary information

Stimuli sample

ANOVA tests results and effect sizes for the target vowel contrasts

Spectral measurements for vowel pairs
