# Supplementary material for: Development of neural perceptual vowel spaces during the first year of life
Source: Sci Rep. 2019 Dec 20;9:19592. doi: 10.1038/s41598-019-55085-y (PMC6925299; doi:10.1038/s41598-019-55085-y)
Supplement: Supplementary file 2 — ANOVA tests results and effect sizes for the target vowel contrasts [file 41598_2019_55085_MOESM2_ESM.pdf]

Development of neural perceptual vowel spaces during the first year of life

Kathleen M McCarthy[*1, 3], Katrin Skoruppa[2], and Paul Iverson[3]

[1]Department of Linguistics, Queen Mary University of London, Mile End Road, London E1 4NS, United Kingdom

[2] Institut des Sciences du Language et de la Communication, Université de Neuchâtel, Rue Pierre-à-Mazel 7, 2000 Neuchâtel, Switzerland

[3]Speech, Hearing and Phonetic Sciences, University College London, Chandler House, 2 Wakefield Street, London, WC1N 1PF, United Kingdom

ANOVA tests results and effect sizes for the target vowel contrasts

Contrast	Sum of squares	*F* d.f (2, 58)	*p*	η_p^2
i-ɪ	99.71	2.17	0.123	0.07
i-ɛ	66.42	1.73	0.186	0.06
i-a	56.21	1.26	0.292	0.04
i-ɒ	47.38	1.10	0.338	0.04
i-ɔ	41.82	1.08	0.346	0.03
i-u	57.49	1.74	0.184	0.06
ɪ-ɛ	157.52	5.17	0.008	0.15
ɪ-a	177.26	3.72	0.030	0.11
ɪ-ɒ	113.99	1.98	0.146	0.06
ɪ-ɔ	125.37	4.33	0.017	0.29
ɪ-u	196.59	5.43	0.006	0.16
ɛ-a	114.29	4.92	0.010	0.15
ɛ-ɒ	265.89	7.32	0.001	0.20
ɛ-ɔ	50.72	1.57	0.321	0.04
ɛ-u	83.27	2.07	0.135	0.06
a-ɒ	237.09	8.56	0.000	0.23
a-ɔ	93.86	1.77	0.183	0.06
a-u	30.71	0.68	0.509	0.02
ɒ-ɔ	128.56	3.21	0.047	0.10
ɒ-u	181.11	3.68	0.031	0.11
ɔ-u	94.49	3.15	0.050	0.10
