# Supplementary material for: Development of neural perceptual vowel spaces during the first year of life
Source: Sci Rep. 2019 Dec 20;9:19592. doi: 10.1038/s41598-019-55085-y (PMC6925299; doi:10.1038/s41598-019-55085-y)
Supplement: Supplementary file 3 — Spectral measurements for vowel pairs [file 41598_2019_55085_MOESM3_ESM.pdf]

Development of neural perceptual vowel spaces during the first year of life

Kathleen M McCarthy[*1, 3], Katrin Skoruppa[2], and Paul Iverson[3]

[1]Department of Linguistics, Queen Mary University of London, Mile End Road, London E1 4NS, United Kingdom

[2] Institut des Sciences du Language et de la Communication, Université de Neuchâtel, Rue Pierre-à-Mazel 7, 2000 Neuchâtel, Switzerland

[3]Speech, Hearing and Phonetic Sciences, University College London, Chandler House, 2 Wakefield Street, London, WC1N 1PF, United Kingdom

Table 2: Spectral measurements for vowel pairs

Contrast	F1 difference (ERB)	*F2 difference* (ERB)	*Spectral difference (Phon)*
i-ɪ	1.19	1.09	1.73
i-ɛ	5.71	2.69	4.19
i-a	6.94	4.10	4.48
i-ɒ	4.97	6.90	4.44
i-ɔ	1.13	8.75	4.06
i-u	0.02	2.81	2.70
ɪ-ɛ	4.52	1.60	2.97
ɪ-a	5.74	3.01	3.53
ɪ-ɒ	3.78	5.81	3.61
ɪ-ɔ	0.07	7.66	3.53
ɪ-u	1.17	1.72	2.25
ɛ-a	1.22	1.41	1.24
ɛ-ɒ	0.74	4.21	2.17
ɛ-ɔ	4.59	6.06	4.01
ɛ-u	5.69	0.11	3.46
a-ɒ	1.97	2.80	1.65
a-ɔ	5.81	4.65	4.21
a-u	6.91	1.29	3.71
ɒ-ɔ	3.84	1.85	3.10
ɒ-u	4.94	4.09	3.63
ɔ-u	1.10	5.95	3.08
